# Harnessing the wisdom of crowds can improve guideline compliance of antibiotic prescribers and support antimicrobial stewardship

**DOI:** 10.1038/s41598-020-75063-z

**Published:** 2020-11-02

**Authors:** Eva M. Krockow, R. H. J. M. Kurvers, S. M. Herzog, J. E. Kämmer, R. A. Hamilton, N. Thilly, G. Macheda, C. Pulcini

**Affiliations:** 1 Department of Neuroscience, Psychology and Behaviour, University of Leicester, Leicester, LE1 7RH UK; 2 Center for Adaptive Rationality, Max-Planck Institute for Human Development, Berlin, Germany; 3 Department of Emergency Medicine, Inselspital University Hospital, University of Bern, Bern, Switzerland; 4 School of Pharmacy, De Montfort University, Leicester, UK; 5 Université de Lorraine, APEMAC, Nancy, France

**Keywords:** Antimicrobials, Policy and public health in microbiology, Infectious diseases, Health care, Diagnosis, Health policy, Health services, Public health

## Abstract

Antibiotic overprescribing is a global challenge contributing to rising levels of antibiotic resistance and mortality. We test a novel approach to antibiotic stewardship. Capitalising on the concept of “wisdom of crowds”, which states that a group’s collective judgement often outperforms the average individual, we test whether pooling treatment durations recommended by different prescribers can improve antibiotic prescribing. Using international survey data from 787 expert antibiotic prescribers, we run computer simulations to test the performance of the wisdom of crowds by comparing three data aggregation rules across different clinical cases and group sizes. We also identify patterns of prescribing bias in recommendations about antibiotic treatment durations to quantify current levels of overprescribing. Our results suggest that pooling the treatment recommendations (using the median) could improve guideline compliance in groups of three or more prescribers. Implications for antibiotic stewardship and the general improvement of medical decision making are discussed. Clinical applicability is likely to be greatest in the context of hospital ward rounds and larger, multidisciplinary team meetings, where complex patient cases are discussed and existing guidelines provide limited guidance.

## Introduction

Antimicrobial overuse is widespread and presents a major public health threat. It promotes the emergence of drug-resistant infections, which—without action—are predicted to incur annual costs of more than 10 million lives by 2050, more than all cancer deaths combined^1^. A particular type of drug resistance, termed antimicrobial resistance (AMR), refers to adaptations of the biological functions of microbes such as bacteria, which can render them less or non-susceptible to antibiotic drugs, meaning that bacterial infections become difficult or even impossible to treat. While AMR develops naturally, the use of antibiotics accelerates this process^2^. In most countries, antibiotic use is legally regulated and requires prescription from a medical professional^3^, yet prescribing choices often fail to meet international guidelines. Current evidence suggests that around 30–40% of antibiotic prescriptions for hospital patients^4,5^ and up to 60% of antibiotic prescriptions in primary care^6^ are inappropriate, although it has to be acknowledged that inappropriate prescribing is often context-dependent and measuring it thus presents a challenge^7^. To improve antibiotic use and preserve drug effectiveness for future generations, a crucial step is therefore to support doctors in their decision making and encourage guideline adherence to curb inappropriate prescribing.

Inappropriate prescribing can manifest itself in different ways. Examples include (1) prescribing antibiotics when they are medically not required (e.g., for viral infections), (2) prescribing an inappropriate type of antibiotics (e.g., broad-spectrum instead of narrow-spectrum antibiotics), or (3) prescribing antibiotics for an inappropriate length of time^8^. With regard to treatment duration, it is probable that longer durations promote the risk of AMR. Nevertheless, many prescribers consistently deviate from shorter treatment recommendations and prescribe longer courses of antibiotics than necessary^9^.

The reasons for overprescribing are manifold—particularly when it comes to choosing appropriate treatment durations^10^. Lack of awareness of existing recommendations and outdated habits based on previous guidelines are a common problem. For example, many prescribers continue to believe that short or unfinished courses of antibiotics may drive resistance, thus prescribing medication for longer than necessary^11^. Additionally, a key reason for the overprescribing of antibiotics is the concern with individual patient risks associated with bacterial infections. Medical doctors typically strive to minimise their patients’ health risks due to an intrinsic motivation to protect the individuals under their care and to avoid litigation or damage to their professional reputation^8^. Hence, in the clinical context, the interests of individual patients often loom larger than the long-term interests of society, which typically include protecting antibiotic drug effectiveness for many generations. Given these strategic properties, antibiotic prescribing has been conceptualised as a challenging social dilemma^12,13^, which may require special decision mechanisms to protect the collective interests and preserve antibiotic effectiveness.

Most previous interventions to curb overprescribing of antibiotics, including unnecessarily long treatment durations, focused on improving the education and training of prescribers or regulating access to antimicrobials. However, considering the mixed results of these interventions’ effectiveness^14^, alternative or additional approaches need to be considered to improve prescribers’ decisions to prescribe an antibiotic treatment and shorten prescribing durations. Relevant research on the improvement of decision accuracy has been conducted in the field of judgement and decision making. One approach includes collecting individual judgements of members belonging to a larger group of decision makers and aggregating these judgements to derive a final, group-based choice.

In line with the popular proverb “two heads are better than one”, the combined knowledge and intelligence of groups, which is also referred to as the “wisdom of crowds” (WoC) or “collective intelligence”, can often produce better choices than individual ones^15^. WoC describes the phenomenon that the aggregated judgement of a group of people is typically better than the judgement made by a randomly drawn member of the group^16–19^. The surprising accuracy of WoC is typically attributed to a crowd’s diversity of errors, which cancel out when combined^17,18^. Crucially, WoC depends on the independence of each crowd member’s opinion. It is therefore different from team-based decision making, where group members discuss their judgements before reaching a joint decision. Indeed, WoC may offer particular benefits in situations characterised by hierarchical social structures (e.g., hospitals contexts with professional distinctions between doctors, nurses and pharmacists), which could affect more traditional team work by discouraging participation of more junior team members^16^.

Empirical evidence shows that WoC approaches may improve decision accuracy across many abstract experimental tasks, including general knowledge tests^20^, memory tasks^21^ and combinatorial problems^22^. Additionally, a growing number of studies have demonstrated the potential of WoC in more applied contexts of medical decision making^23–27^. A recent computer-simulation study^28^, for example, suggested that medical students’ diagnoses for acute medical patients could be improved through an aggregation of two or more opinions. Despite these promising results, the aggregation of group judgements remains a novel approach within the healthcare sector, and requires further testing in the context of specific medical decision problems. With regard to antibiotic prescribing, some studies investigated interactive decision processes of multi-disciplinary treatment teams, and found that the joined judgements of diverse groups of health professionals (e.g., including microbiologists and pharmacists) could improve guideline compliance and cut medication costs^29–31^. However, no research to date has assessed the benefits of non-interactive judgement aggregation of antibiotic prescribing decisions based on a WoC approach. Given the urgent global need of effective antibiotic stewardship, WoC could offer a new approach to addressing the complex issue of overprescribing.

To harness WoC for solving specific decision-making problems, the judgements of all crowd members are collected and aggregated according to a pre-defined crowd rule. Which crowd rule is applicable and performs best depends on the task at hand^18^. For example, while numeric responses can be aggregated using crowd rules such as the arithmetic mean or median, categorical responses require a different approach (e.g., choosing the most frequent response).

In the following, we provide the first application of WoC theory to the problem of antibiotic decision making. Specifically, we investigate whether WoC can improve decisions about antibiotic treatment durations. Drawing on existing survey data containing choices about antibiotic prescription durations made by 787 medical professionals across 15 patient vignettes^9^, we will test three main research questions.

1. Can WoC help to improve prescribers’ compliance with international guidelines?
2. What WoC conditions (e.g., group size and crowd rule) are most effective in achieving prescriber compliance?
3. What are the underlying patterns of prescribing bias?

## Methods

Our analyses are based on previous survey results^9^ (raw data were obtained from the original authors), which assessed prescriber choices of antibiotic treatment durations for 15 hypothetical clinical scenarios (vignettes). Below, we briefly describe the original data collection and sample before detailing our own study design.

### Materials and procedure

The original study used an electronic questionnaire consisting of a section on participant details (e.g., age, professional role and country of residence), and survey questions about antibiotic prescribing choices. The full questionnaire can be found in the supplemental materials of the original article^9^. Specifically, participants were asked to consider a sequence of 15 clinical vignettes, all of which involved bacterial infections. For each vignette, they were required to make a recommendation of treatment duration based on their regular practice (usual recommendations they make to clinicians outside their home department when advising them). An overview of the different vignettes and descriptive statistics of participants’ treatment recommendations is presented in Table 1.

**Table 1:** Overview of clinical vignettes, treatment recommendations and descriptive statistics for participants’ choices of treatment durations.

No.	Brief description of vignette	Prescribing durations recommended by IDSA/SPILF guidelines		Participants’ choices of treatment durations				
		Lower bound (days)	Upper bound (days)	Number of responses	Mean prescribing choice (days)	Standard deviation (days)	Median prescribing choice (days)	Prescribing range (days)
V1	Child or teenager with meningococcal meningitis	5	7	689	8.19	7.22	7	0–180
V2	Patient with acute cholangitis, successfully and rapidly treated by endoscopic biliary drainage	3	3	736	6.81	3.50	7	0–49
V3	Patient with diffuse peritonitis (after surgery with an early adequate source control)	4	7	744	7.46	3.72	7	0–49
V4	Uncomplicated pyelonephritis in an adult woman (if a fluoroquinolone is prescribed)	5	7	759	7.34	2.28	7	0–21
V5	Complicated acute pyelonephritis in an adult woman	10	14	752	12.09	3.83	14	1–60
V6	Patient presenting an acute exacerbation of a severe COPD	5	5	726	6.19	3.37	7	0–60
V7	Outpatient with an uncomplicated pneumonia	5	7	754	6.61	2.24	7	0–49
V8	Patient presenting an uncomplicated catheter-related *Staphylococcus aureus* bacteraemia (without endocarditis and with early removal of the catheter), after negative blood cultures	14	14	752	11.93	4.51	14	2–49
V9	Uncomplicated catheter-related *Klebsiella pneumoniae* bacteraemia (with early removal of the catheter)	7	14	747	9.00	4.86	7	0–90
V10	Uncomplicated *Escherichia coli* vertebral osteomyelitis in a patient without orthopaedic implant	42	42	725	43.61	20.53	42	0–180
V11	Patient presenting a diabetic foot infection, with bone infection, not eligible for surgery	42	84	665	48.63	30.07	42	0–180
V12	Patient with an uncomplicated staphylococcal prosthetic joint infection, managed with 1-stage exchange	42	84	648	57.95	35.17	42	0–365
V13	Patient presenting with an uncomplicated (streptococcal) erysipelas	5	7	703	8.06	2.89	8	3–42
V14	A child (2 years old) with a first episode of acute otitis media	5	5	557	4.72	3.19	5	0–14
V15	An adult with an uncomplicated bacterial maxillary sinusitis	5	7	693	6.86	3.68	7	0–28

*IDSA* Infectious Diseases Society of America, *SPILF* Société de Pathologie Infectieuse de Langue Française, the French Infectious Diseases society.

### Participants

The empirical data were collected between September and December 2016^9^. The study was uncompensated and advertised in newsletters of professional medical networks including ESCMID (European Society of Clinical Microbiology and Infectious Diseases) and SPILF (Société de Pathologie Infectieuse de Langue Française, the French Infectious Diseases society). The sample consisted of expert antibiotic health professionals (e.g., microbiology or infectious diseases specialists), who advised clinicians on antibiotic use at least once per week outside their home department. Following the deletion of participants who had failed to provide any responses to the 15 vignettes, we obtained a data set of 787 participants (417 males and 365 females, five participants did not disclose their sex) out of 1053 who participated in the original survey. Thirty-seven participants were under the age of 30, 504 ranged between 30 and 50 years, 243 were older than 50, and three participants did not disclose their age. The majority of professionals had ten or more years of professional experience. While the sample included participants from 57 countries, most participants were recruited from countries in Western Europe including 156 from France, 128 from the UK, 75 from Spain and 69 from Germany. A detailed overview of sample characteristics is included in the original survey publication^9^.

### Design

**Figure 1:**
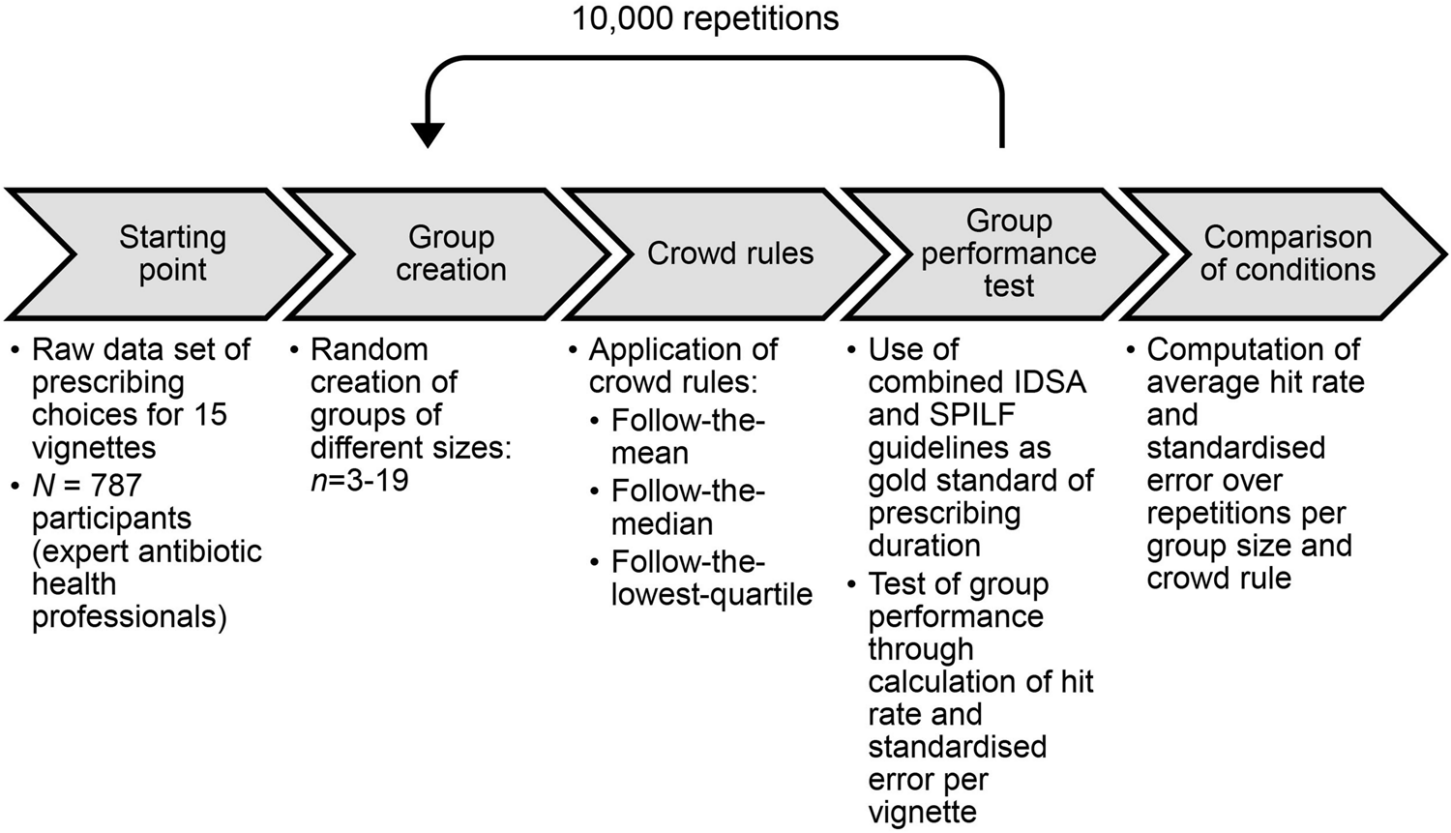
Procedure of wisdom of crowds group simulations. *IDSA* Infectious Diseases Society of America, *SPILF* Société de Pathologie Infectieuse de Langue Française, the French Infectious Diseases society.

We used descriptive statistics to characterise prescribing patterns and ran computer simulations to explore the potential of WoC to increase the number of prescribing recommendations in line with international guidelines (see below for details) and to decrease the prescribing error per vignette. The simulations involved randomly creating prescriber groups from previous survey results^9^, with group sizes ranging between three and 19 prescribers. Prescribers with missing values for the vignettes in question were omitted from the randomly created groups and missing cases were filled up with a new randomly drawn person. A maximum group size of 19 was chosen because even though regular ward rounds usually involve group sizes of five physicians, multidisciplinary team meetings typically involve 10–15 and might even achieve participant numbers up to 19 depending on the topic, meeting objective and the hospital size. Larger group sizes of doctors are, however, unfeasible given typical staffing constraints in most healthcare settings. For each group size, we ran different simulations of data aggregation based on three different crowd rules discussed below. For each group size and crowd rule, we ran 10,000 repetitions. A visual overview of our procedure is presented in Fig. 1.

### Crowd rules

The effectiveness of different crowd rules for numeric responses depends on the presence and direction of bias in the population from which the groups are drawn (e.g., over- vs. underestimation). Symmetrically distributed errors, leading to similar amounts of over- and underestimation^20,32,33^, are best reduced by averaging rules that combine the individual judgements using a central, univariate moment (e.g., arithmetic mean or median). In contrast, in the presence of substantial population bias (i.e., systematic differences between the population average and the correct answer), the performance of crowd rules, such as the mean, is limited by the size of the population bias because such rules, by design, approximate the population bias. Here, different approaches are needed that consider the direction and size of the population bias (e.g., calculating a specific percentile, such as the first quartile of a sample of judgements). In the context of antibiotic prescribing, for example, it is paramount to distinguish between prescribing errors that resulted from overprescribing and underprescribing, respectively.

We tested three different crowd rules to aggregate individual decisions. The first two rules involved following either the mean or the median response, which represent two standard techniques for averaging judgements. The third rule involved following the sample’s lower (i.e., first) quartile.

1. The “Follow-the-mean” rule (F-Mean), involved calculating the mean of all group members’ individual responses and rounding to the nearest integer.
2. The “Follow-the-median” rule (F-Med) involved calculating the median (50th percentile) for all group members’ responses and rounding to the nearest integer. Since the number of prescription days is bounded below (i.e., minimum prescription length is 0 days), we can expect that the distribution of days is right skewed. Hence, the median is expected to result in lower prescription recommendations than the arithmetic mean and thus in lower levels of overprescribing.
3. The “Follow-the-lowest-quartile” rule (F-Quart) involved calculating the lower (i.e., first) quartile (25th percentile) for all group members’ responses and rounding to the nearest integer. For this rule, a minimum group size of four was required, so results will not be displayed for the smallest group size of 3. This rule was included with the specific aim to address the frequently observed bias towards overprescribing.

### Data analysis

To assess the accuracy of prescribing choices, we compared individual responses and the outcomes of each crowd rule simulation against the combined recommendations of infection-specific prescribing durations issued by the Infectious Diseases Society of America (IDSA) and SPILF. The guidelines of these internationally leading organisations on infectious diseases are evidence-based and are widely accepted in the US and in France, and to a lesser extent in other countries when national guidelines are not available, and form the basis of many local hospital guidelines. Acknowledging that these guidelines are not the standard in all countries worldwide, for the purpose of this exploratory study we used their treatment recommendations to define a range of appropriate treatment durations for antibiotic prescribing. For most vignettes, both IDSA and SPILF treatment recommendations entailed a range of possible durations, marked by a lower and an upper bound (see Table 1). In particular, for the lower bound we used the smaller of the two lower bounds (IDSA or SPILF) and for the upper bound we used the larger of the two upper bounds (IDSA or SPILF). For one third of the vignettes (V2, V6, V8, V10 and V14), IDSA and SPILF recommendations specified a single appropriate treatment duration instead of a range. For these, we recorded the upper and lower bounds as identical (i.e., the single recommended duration).

We calculated three types of accuracy. (1) We coded each response that fell within the recommended range of the respective vignette as a “hit” (i.e., indicating a correct response, compliant with the guidelines). Then we calculated the “hit rate”, that is, the proportion of vignettes for which a correct prescribing choice was made. (2) To capture over- vs. underprescribing, we calculated for each response a “signed error”, that is, by how many days the response either overshot the upper bound (coded as a positive error) or undershot the lower bound of the respective range (coded as a negative error); for hits, we coded the error as zero. Hence, a positive signed error indicates overprescribing and a negative signed error underprescribing. Because vignettes ranged widely in their recommended duration (Table 1), we standardised the signed error—to facilitate comparing and summarising across different vignettes—by dividing each value by the centre of the respective recommended range of that vignette (i.e., the arithmetic mean of the lower and upper bound), yielding a “standardised signed error”. Summarising several such values using the arithmetic mean gives the “mean standardised signed error” or, in short, the “standardised bias”, which captures any systematic tendency to over- or underprescribe. (3) To capture the magnitude of prescription errors (i.e., irrespective of whether they represent over- or underprescribing) we calculated the “absolute standardised error”, that is, the magnitude of the standardised signed error (i.e., its absolute value, ignoring the sign). Averaging several such values gives the “mean standardised absolute error” or, in short, the “standardised error”.

## Results

**Figure 2:**
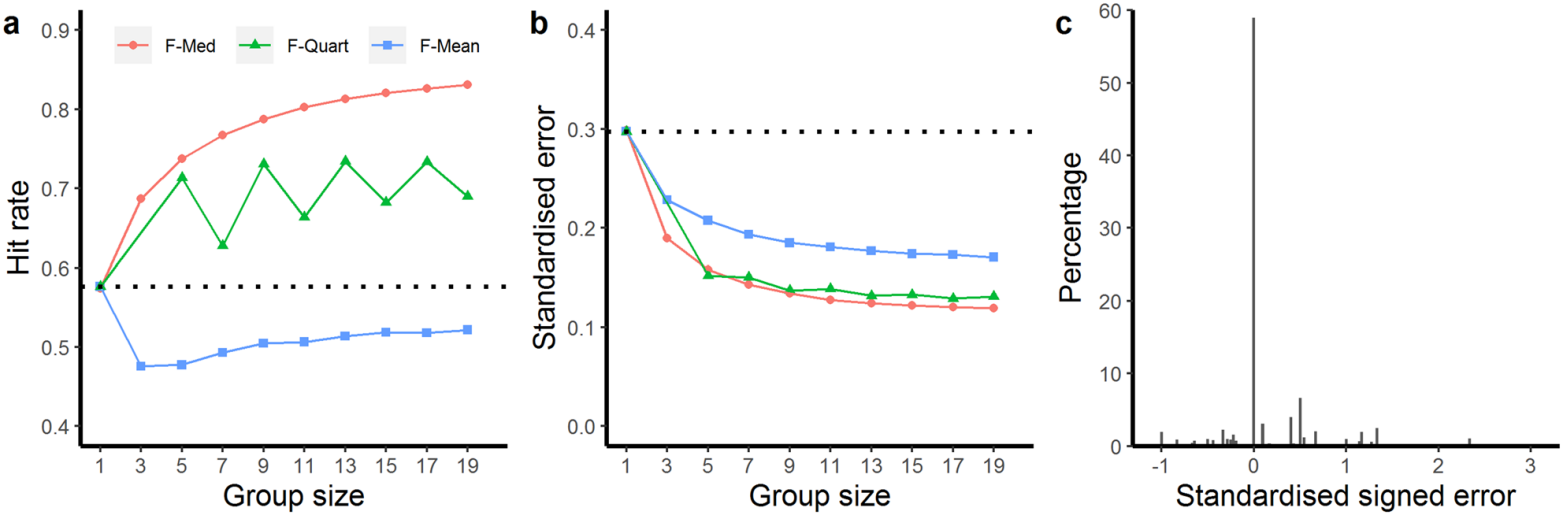
Comparison of (**a**) average hit rates and (**b**) standardised error for the three crowd rules (averaged across all 15 vignettes) for varying group sizes. The x axes display the group sizes, and the y axes show average hit rates (ranging from 0 to 1) and the standardised error, respectively. The dotted lines represent the average hit rate and standardised error for the data of individual prescribers (group size of *n* = 1). (**c**) Shows the standardised signed error for individual prescriber data (group size *n* = 1) pooled across all vignettes.

Figure 2a,b shows the average hit rates and standardised error for different group sizes and crowd rules, averaged across all 15 vignettes. To improve legibility of the results, only odd-numbered group sizes are shown. The different crowd rules varied in their success in improving decision making about antibiotic treatment durations compared to the average individual prescribing duration. Two out of three crowd rules (F-Med and F-Quart) showed marked improvements in hit rates compared to the data of individual prescribers. All three crowd rules led to noticeable decreases in standardised error. Overall, the most successful rule was F-Med (Fig. 2a,b). Indeed, the hit rate already showed a noticeable increase at a group size of only three decision makers. With increasing group sizes, an almost strict increase of hit rate and an almost strict decrease in standardised error was found. Figure 2c shows the standardised signed error for individual prescribers (i.e., group size *n* = 1) across all vignettes. Almost two thirds of prescribing choices had a standardised signed error of 0 and were, therefore, fully compliant with the IDSA and SPILF guidelines (Fig. 2c). Comparing the distributions to the left and right of the 0 value, we observe a larger proportion of the distribution on the right hand side (i.e., positive values), reflecting the overall tendency to overprescribe.

**Figure 3:**
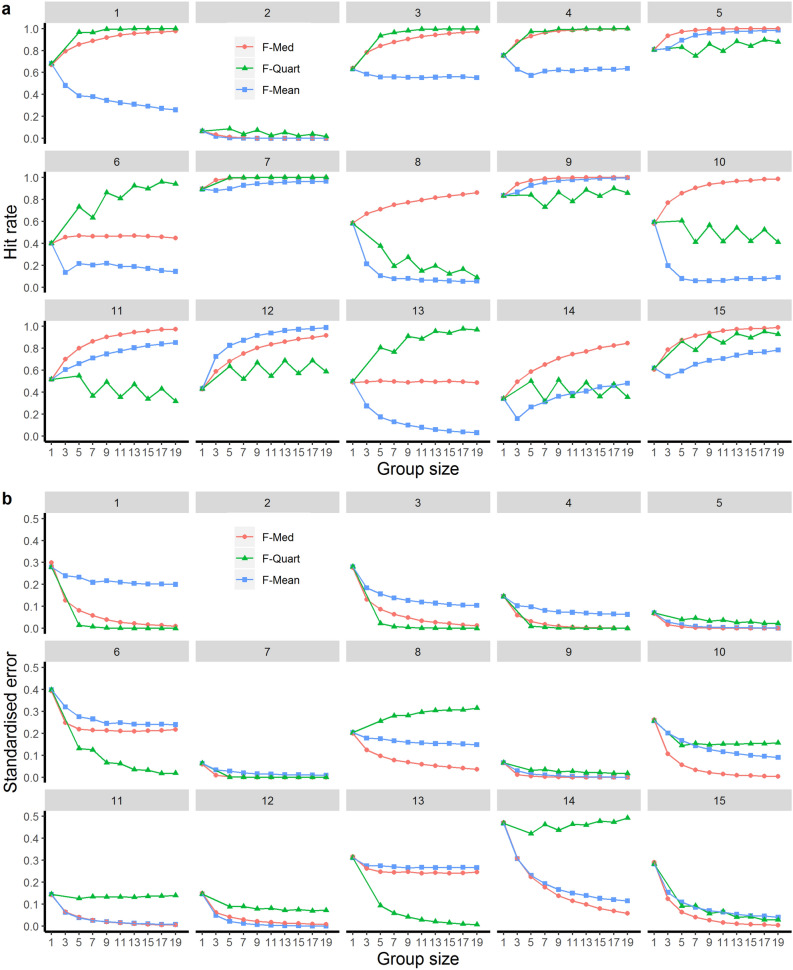
Comparison of hit rates and standardised error for the three crowd rules for varying group sizes per vignette (1–15). The x axes display the group sizes, and the y axes show (**a**) hit rates (ranging from 0–1), and (**b**) standardised error. Note that for Vignette 2 the standardised error exceeded 0.5 (the upper bound of the plotted y-axis); see Supplementary Fig. 1 for the full results of Vignette 2.

Next, we present results specific to each vignette. As shown in Fig. 2a,b, the WoC approaches were mostly successful in improving guideline compliance compared to the treatment choices by individual prescribers. However, Fig. 3 demonstrates that their effects varied across vignettes (again, only the results for odd-numbered group sizes are shown). F-Med was highly successful in increasing hit rates for all vignettes apart from Vignette 2 (acute cholangitis), Vignette 6 (acute exacerbation of a severe COPD) and Vignette 13 (uncomplicated streptococcal erysipelas). Neither F-Mean nor F-Quart (Figs. 2 and 3) performed consistently well in improving guideline compliance of prescribers. Indeed, Fig. 3a demonstrates that, for some vignettes (most notably V1, V8, V10 and V13), hit rates of F-Mean and in some cases F-Quart *decreased* as group size increased.

**Figure 4:**
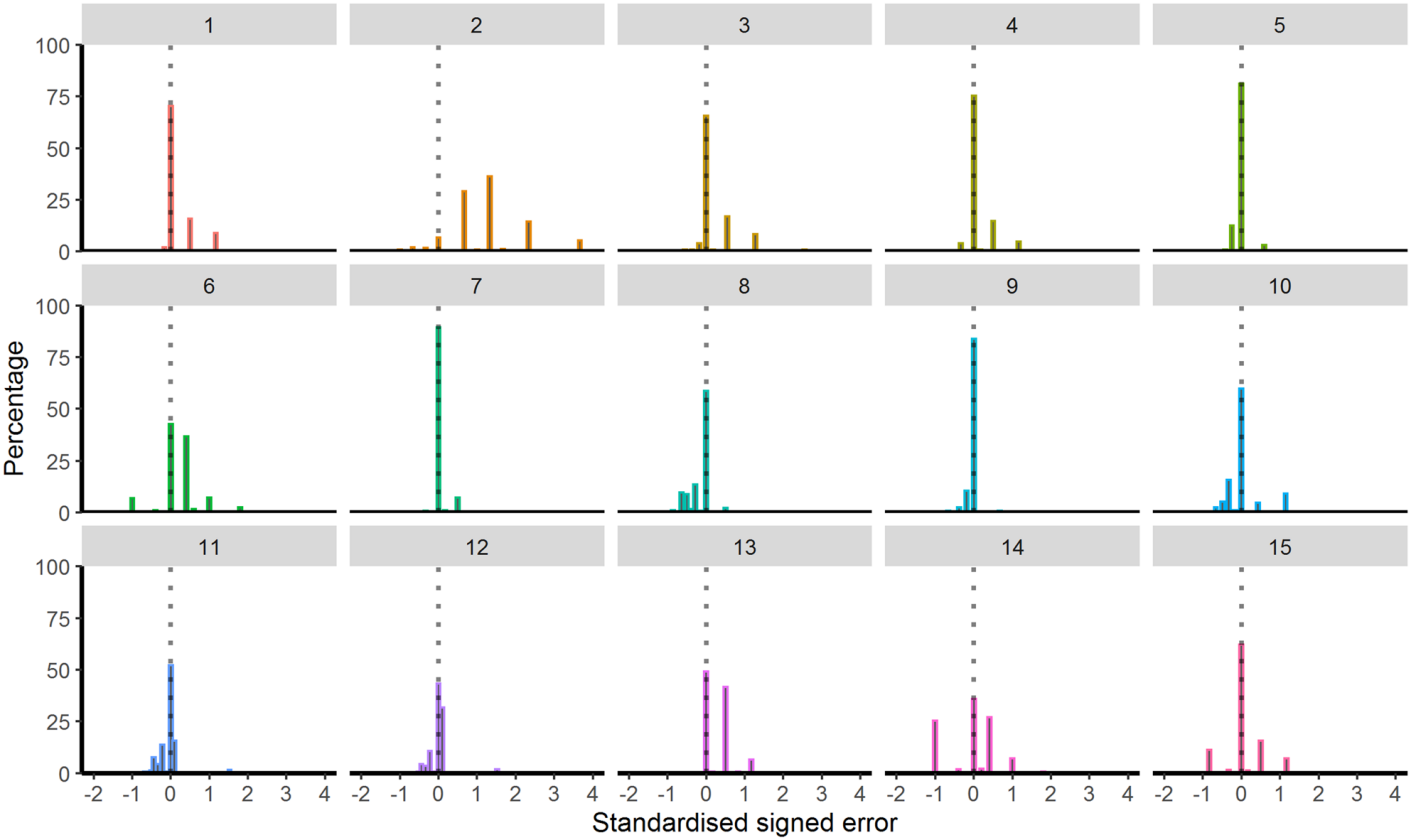
Standardised signed error per vignette when following combined IDSA and SPILF guidelines on prescribing durations. The x axes show the standardised signed error, with 0 indicating that the response fell within the appropriate prescribing range (i.e., no over- or underprescribing). A negative (positive) x-value indicates underprescribing (overprescribing). The y axes show the percentage of responses for each value of standardised signed error.

Finally, Fig. 4 presents the standardised signed error for individual prescribers across the 15 vignettes. With the exception of Vignette 2 (acute cholangitis), the most frequent value for each vignette was 0, indicating that the majority of prescribers chose prescribing durations compliant with the IDSA and SPILF guidelines. Vignette 2 and to a lesser extent Vignettes 6 (acute exacerbation of a severe COPD) and 13 (uncomplicated streptococcal erysipelas) showed a stronger bias towards overprescribing, with 82%, 46% and 45% of treatment recommendations, respectively, falling above the recommended duration. In the case of Vignette 2, most treatment recommendations had a standardised signed error of either 0.67 or 1.33, which corresponds to prescribing 5 and 7 days, respectively.

## Discussion

Our study presents the first application of a WoC approach to the area of antibiotic decision making, thus offering a novel combination of the research on judgement and decision making and the fields of health-related behaviour change and antibiotic stewardship. Re-analysing an extant data set of prescriber choices for antibiotic treatment durations in the context of 15 medical case vignettes^9^, we used computer simulation to randomly create groups ranging between three and 19 prescribers. Following the recommendations of established antibiotic prescribing guidelines (IDSA and SPILF), we subsequently tested whether the aggregated group choices could improve treatment decisions compared to those of individual prescribers. We compared three crowd rules—“Follow-the-mean” (F-Mean), “Follow-the-median” (F-Med) and “Follow-the-lowest-quartile (F-Quart)—and found evidence for the success of aggregating group decisions to improve guideline compliance. The most successful crowd rule was F-Med, which calculated the median prescribing duration (rounded to the closest integer) for each group of prescribers. It improved guideline compliance by increasing hit rates and decreasing the standardised error for 80% of the vignettes. While best guideline compliance was achieved with our maximum group size of 19 prescribers, F-Med produced improvements of both hit rate and standardised error for group sizes of as little as three prescribers. F-Med outperformed F-Mean and F-Quart, both of which produced mixed results.

In fact, a peculiar phenomenon was observed for some vignettes in the context of F-Mean and F-Quart. A number of vignettes (e.g., V1, V8, V10, and V13) showed a decrease in hit rate as group size increased. A possible explanation is that as group size increases, the crowd rule output becomes less variable; in the extreme case of using the whole population, the crowd rule will always output the same collective judgement (by necessity). If the population is unbiased, then having less variance (as group size increases) is of benefit, because the crowd’s judgement is increasingly more likely to land near the population average and thus to be within the range of appropriate prescribing. However, for vignettes with noticeable bias, low variance is—somewhat ironically—a disadvantage, because with increasing group size it becomes increasingly unlikely that the crowd judgement will—by sheer luck (i.e., sampling error)—land within the correct range.

A reason for the comparative success of F-Med, is the robustness of the median^34,35^, which can be explained by its high “breakdown point”. This measure refers to the proportion of observations that would need to be moved into the same direction (e.g., higher value) so that the estimator (e.g., the median) starts to move itself. For the median this breakdown point is 50%, which means that more than half of the chosen antibiotic prescribing durations would need to move in the same direction (either all over- or all underprescription) before the median starts to shift. By comparison, the mean is much more easily influenced and can be distorted by a single extreme response.

Despite the overall success of F-Med, however, three exceptions were identified amongst the 15 vignettes used in the antibiotic prescribing survey: Vignettes 2 (acute cholangitis), 6 (acute exacerbation of a severe COPD) and 13 (uncomplicated streptococcal erysipelas). Vignette 2 showed no improvement in either hit rate or standardised error when individual choices were aggregated at the group level. To a lesser extent, Vignette 6 and Vignette 13 were also exceptions. At a maximum group size of 19 decision makers, their likelihood of choosing a guideline-compliant treatment duration was only around 50%. The poor performance of F-Med in the context of these vignettes could be attributed to the particularly high levels of overprescribing found in the individual-level data. This is in contrast with most other vignettes, which showed more equal proportions of under- and overprescribing. The biased distributions for Vignettes 2, 6, and 13 likely rendered these contexts unsuitable to averaging techniques such as calculating the median. Consistent with this claim, the F-Quart rule—which by design corrects for overprescribing—outperformed F-Med for these three vignettes.

The reason for variations in the patterns of over- vs. underprescribing in the individual-level responses, however, is more difficult to explain. The large levels of overprescribing in Vignettes 2, 6 and 13 may have been due to participants’ misunderstanding the clinical scenario described by the vignette. Another, perhaps more likely, possibility is that the IDSA and SPILF guidelines for these vignettes are less established, thus accounting for a general lack of knowledge about appropriate treatment durations. Indeed, it is important to note that only limited research on optimal prescribing durations has been conducted to date, leaving antibiotic treatment durations open to debate^36^. Recent clinical trials, for example, have suggested that traditional courses of antibiotics could be shortened without compromising patient safety^37, 38^, but this latest evidence has not yet been translated into guidelines. Hence, even though IDSA and SPILF guidelines are used widely internationally, their recommendations are by no means undisputed^39,40^. This also leaves prescribers more vulnerable to behavioural biases. In the case of Vignette 2, for example, a closer analysis of the participants’ choices demonstrated a bias towards the prescription of treatments with durations of either 5 or 7 days (or multiples thereof). This 5/7-day bias has previously been reported in the context of antibiotic prescribing^40–43^ and is likely due to an inherent cultural tendency to use simple units of measurement (e.g., five fingers per hand and 7 days per week), which simultaneously serves as a memory aid. While the bias may not always present a problem, it could be particularly damaging in the cases of short-term bacterial infections, which can be treated successfully with only 2 or 3 days of medication.

Given our identification of several exceptions in the clinical cases considered for our analysis, it is important to identify and predict criteria of future patient cases that make them suitable for a WoC approach and specifically the “Follow-the-median” rule, which was found to be the most successful rule for the current data set. Qualitative follow-up research (e.g., based on verbal protocol analysis) could explore the individual decision-making processes for the 15 original vignettes or additional patient cases in more depth and thereby identify indicators that either increase or decrease the applicability of WoC approaches to specific clinical decision dilemmas. A consequently increased understanding of the reasoning processes underlying recommendations for prescribing durations could enable the more targeted application of WoC data aggregation methods.

Our results have important implications for the field of medical decision making and, in particular, the challenging task of antibiotic stewardship. Previous behaviour change interventions to decrease unnecessary antibiotic prescribing relied on either restrictive techniques to deter overprescribing of antibiotics or persuasive approaches to encourage a more conscious decision process guided by better information about the recommended diagnostic and therapeutic decision process and the societal risk of AMR. Unfortunately, the effectiveness of these approaches varied^14,44^. Against this background, our evidence for the potential of crowd-based decision making without the need of introducing large-scale educational programmes is highly promising.

In addition to improved prescribing, the WoC approach provides another potential benefit. Group-based judgements could make an important contribution towards relieving individual doctors from patient pressures and fears of litigation. Previous literature^8^ has highlighted the prescribers’ common worry about poor patient outcomes, which often leads them to err on the side of overprescribing antibiotics. Introducing WoC-based procedures for making treatment decisions could be an important step towards diffusing individual prescriber responsibility and providing a more protected decision environment with less contextual drivers of overprescribing. Follow-up research is needed to test this additional benefit in real-life clinical decision contexts and investigate the wider applicability of WoC-based aggregation techniques.

### Addressing other aspects of antibiotic decision making

The present study focused on prescriber decisions about the *durations* of antibiotic treatment. This had the benefit of being able to draw on a set of numeric interval data, thus allowing for a variety of different mathematical crowd rules to be tested. However, the problem of antibiotic overprescribing is not limited to excessive prescribing durations. Additional challenges include, for example, misguided choices to prescribe antibiotics in the absence of bacterial infections or choices to prescribe inappropriate types of antibiotics^8^. Both of these cases of inappropriate prescribing are examples of a different problem format, which requires categorical responses from the decision maker rather than numerical ones. A consequence of the different format is that applicable crowd rules are likely to differ. In fact, none of the crowd rules investigated in the current study would translate to a categorical problem format. Future research therefore needs to test the applicability of WoC in the context of other antibiotic decision aspects and identify what crowd rules and group sizes are most helpful for improving decision making in those situations.

### Studying a variety of prescriber samples

The current study was conducted using a sample of infection experts (e.g., microbiologists or infectious disease specialists), who regularly give advice to less experienced colleagues. It is therefore likely that the overall performance of our sample was higher (i.e., better guideline compliance and overall shorter prescribing durations) than what could be expected from a mixed sample of frontline, non-specialist prescribers. It is possible that overprescribing is more frequent in samples with lower expertise in the field, thus necessitating a more conservative crowd rule like F-Quart to compensate for this selection bias. To investigate such differences, more research is needed on prescribing patterns of different sub-populations of health professionals.

### Maximising real-life applicability

An important prerequisite for more applied research on WoC in clinical decision making is to identify real-life choice contexts that fulfil the key assumptions of WoC and thus provide a suitable decision environment to enable successful WoC applications. The main assumption outlined in the Introduction is a diverse crowd of decision makers with independent judgement errors^18,45^. Below, we discuss this assumption in the context of three common prescribing contexts: outpatient consultations, hospital ward rounds, and multidisciplinary team meetings.

#### Outpatient consultations

Outpatient appointments are typical for the primary care sector as well as treatment of long-term, non-acute patients in secondary and tertiary care. Appointments are typically led by single clinicians. Given the hectic and pressurised choice environments of many clinical healthcare settings^46,47^, individual prescribers are frequently pressed to make instantaneous choices that prevent them from consulting colleagues. The feasibility of consulting “crowds” of decision makers in such outpatient consultations is therefore questionable. Rather than aggregating different prescriber opinions in real-time, other physicians’ antibiotic choices could be discussed as retrospective multidisciplinary team and case-review meetings, or be recorded and communicated to those who frequently overprescribe. Previous research has confirmed the potential of providing such feedback in primary care^10^.

#### Ward rounds

Hospital ward rounds are routinely conducted by medical teams consisting of around three to five medical staff (consultants, junior doctors, and students), pharmacists, nurses, and allied-health professionals. Given that our results show that WoC can improve decisions about antibiotic treatment durations in groups as small as three decision makers, ward rounds could provide viable settings to trial WoC-based decision aggregation and compare its effectiveness with decisions reached through joint team discussions. A potential challenge in smaller groups are disproportionate influences of senior group members. In the hierarchical decision-making contexts of many traditional hospitals, junior doctors often feel compelled to agree with their supervisors’ judgements^48^, and pharmacists may have less authority than medical doctors^49^. In order to minimise this bias and maximise the benefits of WoC during ward rounds, health professionals’ judgements would therefore need to be collected individually and anonymously. Furthermore, whenever teams pre-commit to using the median prescription length as the group’s final judgement, then the robustness of the median (see the earlier discussion on this) prevents an unduly high influence of, say, one senior group member in a group together with two other, less senior group members.

#### Multidisciplinary team meetings

Multidisciplinary team meetings refer to larger meetings of up to 10–15 different specialist doctors, nurses, pharmacists, and other health professionals. Given our findings of improved decision accuracy with increased group sizes, multidisciplinary team meetings are likely to yield the most reliable improvements of prescribing choices when using WoC. Additionally, the patient cases discussed during multidisciplinary team meetings might be most appropriate for WoC-based decision making. These typically include complex, long-term cases, where adequate evidence for the optimal duration of therapy is unavailable and existing guidelines fail to provide clear treatment recommendations. It is in those cases that the diverse experience of crowds could provide the most useful insights. To preserve such diversity at multidisciplinary team meetings, it is crucial to check the absence of institutional prescribing biases^46,50^. Helpfully, hospitals and other health care institutions in many countries are characterised by frequent turnover of staff (particularly junior doctors) based on nationally regulated rotations of doctors. These regular changes in the composition of prescriber teams therefore guarantee some level of diversity.

To test feasibility and acceptability of WoC approaches in medical decision contexts, more applied research is necessary. Specifically, clinical intervention studies could help to evaluate the results of WoC-based decision procedures and trial technical tools (e.g., anonymous voting tools) to support the process.

## Conclusions

Collecting and aggregating independent judgements of medical prescribers in line with the concept of the wisdom of crowds appears to be a promising decision technique to improve guideline compliance in the context of antibiotic decision making and reduce antibiotic overuse. Our simulation study provided evidence for the respective benefits of different group aggregation techniques to reduce inappropriate choices about antibiotic prescribing durations. Overall, using the median group judgement appears to yield the largest benefits. While decision accuracy increased with group size, improvements were found for groups as small as three prescribers. Follow-up research is necessary to explore the potential of collective decision making across a larger variety of prescriber samples and decision contexts. Real-life applicability needs to be tested and is likely to be greatest for hospital ward rounds and larger, multidisciplinary team meetings, where complex patient cases are discussed and existing guidelines provide limited guidance.

## Supplementary information

Supplementary Figure 1.
