# Supplementary material for: Harnessing the wisdom of crowds can improve guideline compliance of antibiotic prescribers and support antimicrobial stewardship
Source: Sci Rep. 2020 Nov 2;10:18782. doi: 10.1038/s41598-020-75063-z (PMC7608639; doi:10.1038/s41598-020-75063-z)
Supplement: Supplementary file 1 — Supplementary Figure 1. [file 41598_2020_75063_MOESM1_ESM.docx]

**Harnessing the wisdom of crowds can improve guideline compliance of antibiotic prescribers and support antimicrobial stewardship**

Krockow, E.M., Kurvers, R.H.J.M., Herzog, S.M., Kämmer, J.E., Hamilton, R.A., Thilly, N., Macheda, G., & Pulcini, C.

**
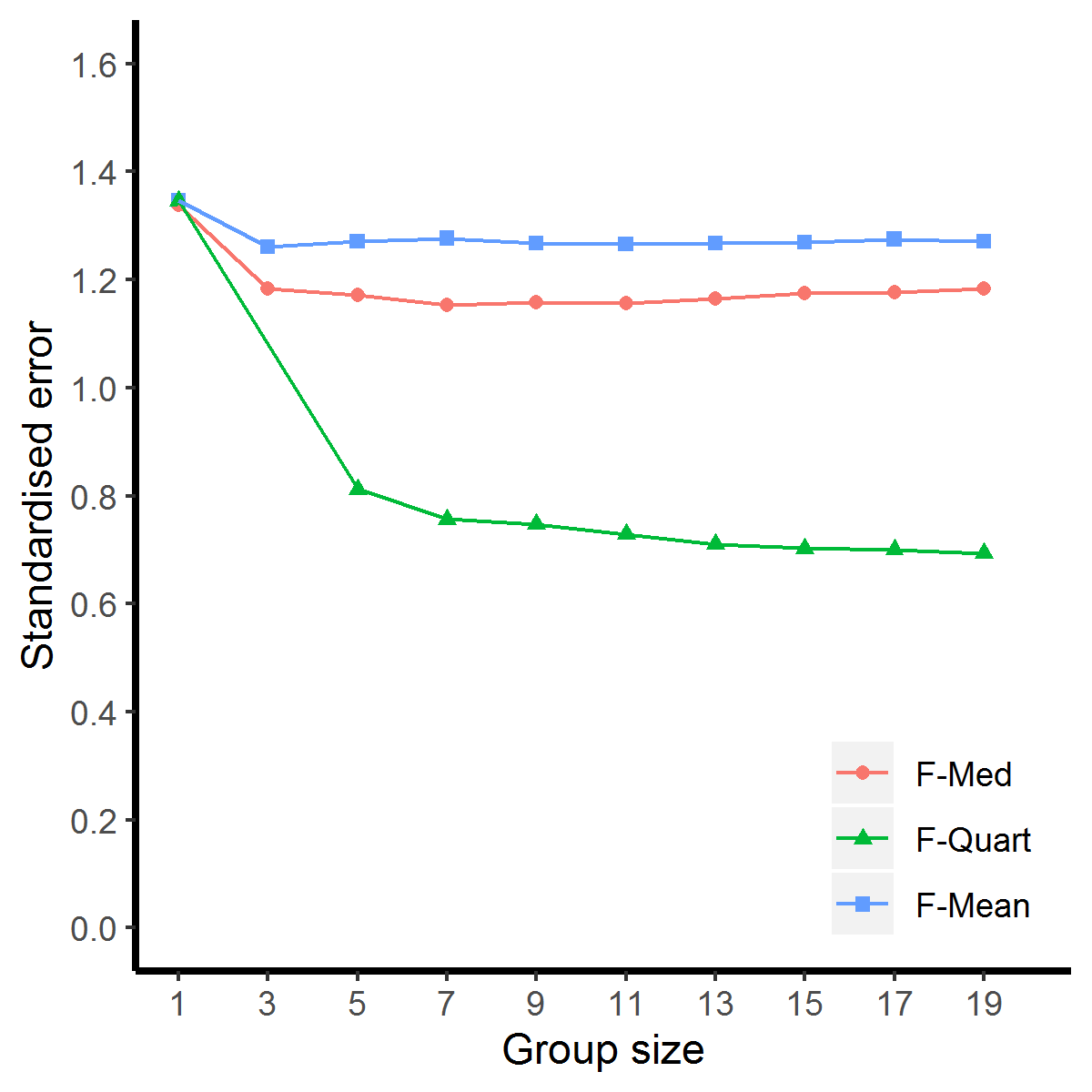
Supplementary Material**

*Supplementary Figure 1.* Comparison of standardised error for the three crowd rules and varying group sizes for Vignette 2 (acute cholangitis). The x axis displays the group sizes and the y axis shows the standardised error.
